# Population-based SEER trend analysis of overall and cancer-specific survival in 5138 patients with gastrointestinal stromal tumor

**DOI:** 10.1186/s12885-015-1554-9

**Published:** 2015-07-30

**Authors:** Ulrich Güller, Ignazio Tarantino, Thomas Cerny, Bruno M. Schmied, Rene Warschkow

**Affiliations:** 1 Division of Medical Oncology & Hematology, Kantonsspital St. Gallen, CH-9007 St. Gallen, Switzerland; 2 University Clinic for Visceral Surgery and Medicine, University Hospital Berne, 3010 Berne, Switzerland; 3 Department of General, Abdominal and Transplant Surgery, University of Heidelberg, 69120 Heidelberg, Germany; 4 Department of Surgery, Kantonsspital St. Gallen, 9007 St. Gallen, Switzerland; 5 Institute of Medical Biometry and Informatics, University of Heidelberg, 69120 Heidelberg, Germany

**Keywords:** Gastrointestinal stromal tumors (GIST), Surveillance, Epidemiology and End Results (SEER) database, Trend analysis, Gastric GIST

## Abstract

**Background:**

The objective of the present population-based analysis was to assess survival patterns in patients with resected and metastatic GIST.

**Methods:**

Patients with histologically proven GIST were extracted from the Surveillance, Epidemiology and End Results (SEER) database from 1998 through 2011. Survival was determined applying Kaplan-Meier-estimates and multivariable Cox-regression analyses. The impact of size and mitotic count on survival was assessed with a generalized receiver-operating characteristic-analysis.

**Results:**

Overall, 5138 patients were included. Median age was 62 years (range: 18–101 years), 47.3 % were female, 68.8 % Caucasians. GIST location was in the stomach in 58.7 % and small bowel in 31.2 %. Lymph node and distant metastases were found in 5.1 and 18.0 %, respectively. For non-metastatic GIST, three-year overall survival increased from 68.5 % (95 % CI: 58.8–79.8 %) in 1998 to 88.6 % (95 % CI: 85.3–92.0 %) in 2008, cancer-specific survival from 75.3 % (95 % CI: 66.1–85.9 %) in 1998 to 92.2 % (95 % CI: 89.4–95.1 %) in 2008. For metastatic GIST, three-year overall survival increased from 15.0 % (95 % CI: 5.3–42.6 %) in 1998 to 54.7 % (95 % CI: 44.4–67.3 %) in 2008, cancer-specific survival from 15.0 % (95 % CI: 5.3–42.6 %) in 1998 to 61.9 % (95 % CI: 51.4–74.5 %) in 2008 (all P_Trend_ < 0.05).

**Conclusions:**

This is the first SEER trend analysis assessing outcomes in a large cohort of GIST patients over a 11-year time period. The analysis provides compelling evidence of a statistically significant and clinically relevant increase in overall and cancer-specific survival from 1998 to 2008, both for resected as well as metastatic GIST.

## Background

Gastrointestinal stromal tumors (GIST) are the most frequent mesenchymal malignancies of the gastro-intestinal tract. The origin of GIST is the cell of Cajal, which is the pace-maker cell located between the circular and longitudinal muscle layer along the gastro-intestinal tract and is responsible for the gastro-intestinal motility. GIST occur most frequently in the stomach and small bowel, other locations such as esophagus, colon, rectum and extravisceral locations are rare.

For many decades surgery was the only efficient treatment modality for GIST. However, despite complete resection, the high recurrence rate remained an unsettling problem. The use of chemotherapy or radiation was proven to be largely ineffective [1]. However, over the past 15 years substantial improvements were made in the understanding of the pathogenesis and treatment of GIST. Around the change of millennium physicians began to understand that GIST are a result of a KIT or PDGFR mutation and more importantly, that the resulting mutated KIT or PDGF receptor could be blocked by the tyrosine kinase inhibitor imatinib. This targeted agent, which previously had a tremendous success in treating chronic myeloid leukemia by blocking the ABL-kinase of the *BCR-ABL* fusion protein, was now also applied in this solid tumor entity. Imatinib was first used in a female patient with a metastatic GIST, who was unsuccessfully treated with different chemotherapies [2]. After 4 weeks of imatinib treatment, a phenomenal response was seen on PET scan. Since then, many studies including several randomized trials have been performed using imatinib in non-metastatic [3, 4] and metastatic GIST [5, 6].

However, it remains unknown whether improvements in understanding and management of GIST patients have resulted in relevant patient benefits on a population-based level. Therefore, the primary objective of the present analysis was to assess whether overall and cancer-specific survival of GIST patients have improved over a 11-year time period.

## Methods

### Cohort definition

The recent ASCII text data-version of the Surveillance, Epidemiology, and End Results (SEER) Program of the National Cancer Institute in the United States, covering approximately 28 % of cancer cases in the United States, was the source of present population-based analysis [7]. SEER data were collected and reported using data items and codes as documented by the North American Association of Central Cancer Registries (NAACCR) [8]. Primary cancer site and histology were coded according to criteria in the third edition of the International Classification of Diseases for Oncology (ICD-O-3) [9].

**Fig. 1:**
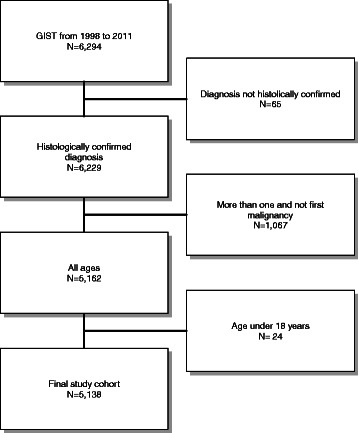
Patient selection

GIST patients were identified by the primary sites esophagus, stomach, small intestine, colon, rectum, appendix, peritoneum and the codes “8935” and “8936” for ICD-O-3 histology. Patients diagnosed at autopsy or by death certificate only as well as patients without histological confirmation were excluded (NAACCR Items 490 and 2180). Patients with other SEER reportable cancers were excluded unless the GIST was the first diagnosed malignancy (NAACCR Item 380) in order to use the cancer-specific survival. Patients with pediatric GIST (n = 24) were excluded from the analysis (Fig. 1). Size was coded as a continuous variable in mm. Five patients with GIST sizes exceeding 70 cm were excluded from analyses involving GIST size.

### Statistical analysis

Statistical analyses were performed using the R statistical software (www.r-project.org). A two-sided p-value < 0.05 was considered statistically significant. Continuous data are expressed as median and interquartile range (IQR). After descriptive analysis, survival was assessed by Kaplan-Meier analysis. Overall and cancer-specific survival were the designated endpoints. For analysis of overall survival, the time from diagnosis until the end of the follow-up was used together with the information whether a patient died or not. For cancer-specific survival, cancer-associated deaths were counted for the estimation of the cancer-specific survival whereas other deaths unrelated to GIST were censored. The censoring was based on the coding of these endpoints in the SEER database (alive, cancer-associated death, other death). P-values were computed using Cox-regression and likelihood-ratio-tests. To assess the association between GIST size and survival, locally weighted scatterplot smoothing (LOWESS)-Regression was performed [10]. To analyze the predictive value of the continuous variables size and mitotic rate for survival, a generalized receiver-operating characteristic (ROC)-methodology for survival analysis was applied [11]. Sensitivity and 1-specificity for prediction of one-year survival were simultaneously plotted as ROC-curves and the area under the curve (AUC) was estimated. Mitotic count was systematically recorded after 2009, therefore only one year survival rates were computable. For each distinct value of mitotic count and size, the pairs of ‘true positives’ (number of patients for whom death was predicted and who died) and ‘false positives’ (number of patients for whom death was predicted and who survived) are displayed [11]. These pairs form the receiver-operating characteristic (ROC)-curve. The area under the curve (AUC) of a perfect predictor would have an AUC of 1 and the ROC-curve would have an ROC plot along the left side and the top of the graph. For prediction due to chance, the AUC is 0.5 and the ROC-curves are on the diagonal line (“chance diagonal”) [12]. The statistically optimal cut-off value was estimated by maximizing the Youden index (computed as Sensitivity + Specificity-1). Multivariable survival analyses were done using Cox regression analyses. The proportional hazard assumption was tested by scaled Schoenfeld residuals and by inspection of the hazard ratio (HR) plots [13]. For trend analysis, Spearman’s rho was applied. Extrapolation of survival rates was based on the covariate vector for the year of diagnosis modeled as a factorial variable in Cox regression.

### Ethics statement

This study was based on public use de-identified data from the SEER database and did not involve interaction with human subjects or use personal identifying information. The study did not require informed consent from the SEER registered cases and the authors obtained Limited-Use Data Agreements from SEER.

## Results

### Patient characteristics

Overall 5138 patients diagnosed with GIST between 1998 and 2011 in one of the regions covered by SEER were eligible for the present analysis (Fig. 1)*.* The median follow-up in our patient cohort was 37 months (interquartile range: 14 to 74 months). The median age was 62 years (interquartile range 52 to 73 years) with a range of 18 to 101 years, 47.3 % were female, 68.8 % Caucasians. GISTs were located in the stomach in 58.7 % and small bowel in 31.2 %. All other locations were rare (Table 1). Lymph node metastases were found in 5.1 %, distant metastases in 18.0 % of all patients (Table 1). Median size of the GIST was 7.0 cm (interquartile range 4.5 to 11.8 cm) with a range from 0.2 to 70 cm.

**Table 1:** Patients’ characteristics

Variable	Category	All GIST
		(*N* = 5138)
Location	Stomach	3018 (58.7 %)
	Small intestine	1603 (31.2 %)
	Other:	517 (10.1 %)
	• Esophageal	29 (0.6 %)
	• Colon	139 (2.7 %)
	• Rectum	172 (3.3 %)
	• Appendix	3 (0.1 %)
	• Peritoneum	174 (3.4 %)
Size categories	<5 cm	1280 (24.9 %)
	5 cm–9.9 cm	1678 (32.7 %)
	10 cm+	1471 (28.6 %)
	Unknown	709 (13.8 %)
Size (cm)	Median [IQR]	7.0 cm [4.5 to 11.8 cm]
	Range	0.2–70 cm
N stage	N−	4071 (79.2 %)
	N+	264 (5.1 %)
	NX	803 (15.6 %)
Mitotic Count^a^	<2 per 50 HPF	397 (7.7 %)
	2–5 per 50 HPF	171 (3.3 %)
	>5 per 50 HPF	159 (3.1 %)
	Unknown	4411 (85.9 %)
Surgery of primary	No surgery of primary tumor	865 (16.8 %)
	Surgery of primary tumor	4263 (83.0 %)
	Unknown	10 (0.2 %)
Metastatic disease	M0	4211 (82.0 %)
	M1:	927 (18.0 %)
	−M1, no surgery of metastasis	−763 (14.8 %)
	−M1, surgery of metastasis	−139 (2.7 %)
	−M1, surgery of metastasis	−25 (0.5 %)
	unknown	
Year	1998 to 2002	1120 (21.8 %)
	2003 to 2005	1195 (23.3 %)
	2006 to 2008	1227 (23.9 %)
	2009 to 2011	1596 (31.1 %)
Gender	Male	2709 (52.7 %)
	Female	2429 (47.3 %)
Age	<50	1060 (20.6 %)
	50–64	1805 (35.1 %)
	65–79	1682 (32.7 %)
	80+	591 (11.5 %)
Ethnicity	Caucasian	3536 (68.8 %)
	African-American	920 (17.9 %)
	Other/Unknown	682 (13.3 %)
Marital status	Married	3008 (58.5 %)
	Single	832 (16.2 %)
	Other/Unknown	1298 (25.3 %)
Cause of death	Alive	3545 (69.0 %)
	Dead from cancer	1080 (21.0 %)
	Dead not from cancer	513 (10.0 %)
Follow-up (months)	Median [IQR]	37.0 [14.0 to 74.0]

^a^ Mitotic count systematically recorded after 2009

### Univariable survival analysis

**Fig. 2:**
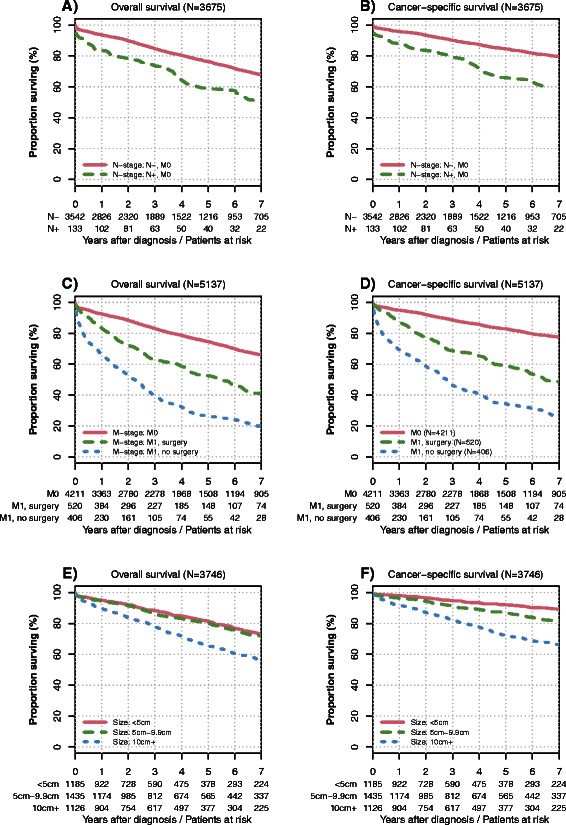
Univariate survival analysis. The upper two plots display the Kaplan-Meier curves for overall (panel **a**) and cancer-specific survival (panel **b**) in cM0 patients with and without lymph node metastases (*P* < 0.001). Panel (**c**) and (**d**) display the Kaplan-Meier curves in the overall cohort comparing patients with non-metastatic and with metastatic GIST who did and did not undergo primary tumour surgery (*P* < 0.001 for all comparisons). Panel (**e**) and (**f**) display the Kaplan-Meier curves for non-metastatic GIST patients according to different primary tumour sizes (<5 cm vs. 5 to 9.9 cm: P = 0.360 for overall survival, all other comparisons: P < 0.001). Numbers of patients at risk are given below the x-axis

At the end of follow-up 3545 (69.0 %) patients were alive, 1080 (21.0 %) died from GIST and 513 (10.0 %) died due to reasons which were not associated with the GIST according to the coding in the SEER database. In patients with non-metastatic GIST lymph node metastases were associated with a significantly decreased overall and cancer-specific survival (P < 0.001, Fig. 2 panel a and b). Overall and cancer-specific survival was significantly decreased in patients with metastatic GIST and further so in patients without surgery of the primary tumor (P < 0.001, Fig. 2 panel c and d). Larger tumors were associated with significantly worse survival: Five-year overall survival rates were 81, 80 and 65 % (P < 0.001) in GIST tumors <5 cm, 5–9.9 cm and = > 10 cm, respectively. Five-year cancer-specific survival rates were 92, 87 and 72 % in the respective size categories (P < 0.001, Fig. 2 panel e and f).

### Tumor size and mitotic count

**Fig. 3:**
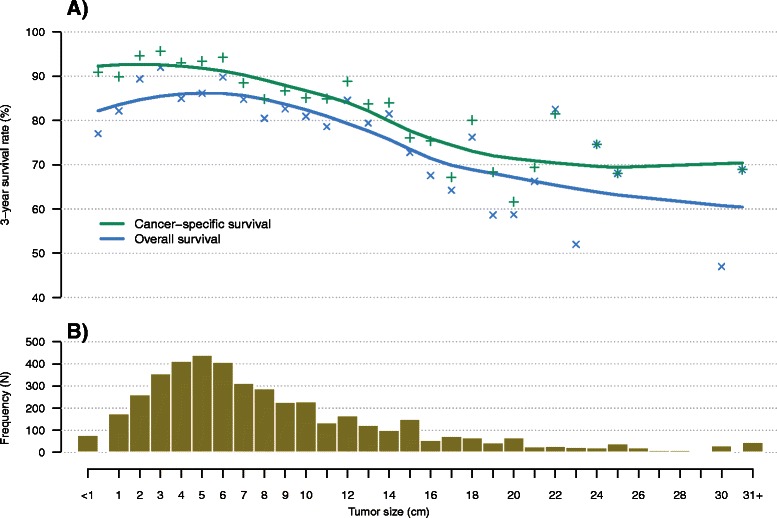
Size of GIST and survival in cM0 GIST patients. Panel (**b**) displays the size distribution of cM0 GIST patients (*N* = 3746). For each size category, overall and cancer-specific survivals were computed (panel **a**)

Figure 3 displays the GIST size (panel b) and its association with overall and cancer-specific survival (panel a) in patients with non-metastatic disease. The size distribution peaks at 5 cm. For sizes exceeding 8 cm a marked decrease in overall and cancer-specific survival was observed for non-metastic GIST patients.

**Fig. 4:**
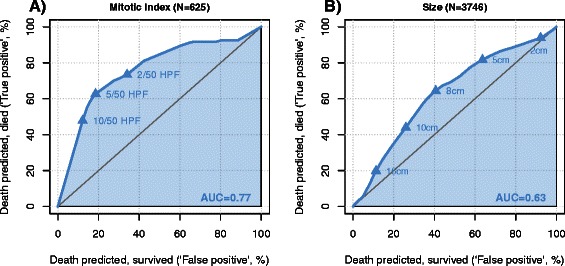
Predictive value of mitotic count and size for one year cancer-specific survival in cM0 GIST patients. On panel (**a**), the predictive value of mitotic count for one year cancer-specific survival is depicted. On panel (**b**), the predictive value of the size of the GIST is demonstrated

**Fig. 5:**
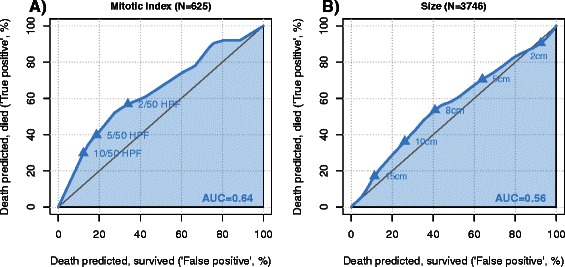
Predictive value of mitotic count and size for one year overall survival in cM0 GIST patients. On panel (**a**), the predictive value of mitotic count for one year overall survival is depicted. On panel (**b**), the predictive value of the size of the GIST for one year overall survival is demonstrated

Figure 4 displays the predictive value of mitotic count (panel a) and tumor size (panel b) for one-year cancer-specific survival using the ROC-methodology. The impact of size on survival is lower compared to the mitotic count (area under the curve of 0.63 compared to 0.77). The statistically optimal (defined as maximal Youden index) cut-off value of mitotic count was 5 in 50 high power fields (HPF). For GIST size, the statistically optimal cut-off is 8 cm. Similar results were obtained for overall survival (Fig. 5). The predictive value of mitotic count (panel a) was higher than the predictive value of the tumor size (panel b). The impact on overall survival was lower than on cancer-specific survival considering the lower area under the curve observed for mitotic count and for tumor size.

### Multivariable survival analysis

In multivariable analysis of overall survival GIST location other than stomach and small bowel (hazard ratio (HR) 1.30, P = 0.002), tumor size above 10 cm (HR 1.63; *P* < 0.001), presence of distant (HR 2.03; P < 0.001) and lymph node metastases (HR 1.47; P = 0.001), older age (*P* < 0.001), single marital status (HR 1.38; P < 0.001), and African-American ethnicity (HR 1.22; P = 0.002) were associated with worse overall survival, whereas patients undergoing primary tumor excision (HR 0.49; P < 0.001), female patients (HR 0.70; P < 0.001), and patients during later time periods (P < 0.001) had significantly improved overall survival (Table 2). Similar results were obtained for the cancer-specific survival except for African-American ethnicity (HR 1.08; P = 0.058) (Table 2)*.*

**Table 2:** Univariable and multivariable analysis of overall and cancer-specific survival

		Overall survival				Cancer-specific survival			
Covariates		Unadjusted ^a^		Cox regression, full model ^b^		Unadjusted ^a^		Cox regression, full model ^b^	
		HR (95 % CI)	*p* ^c)^	HR (95 % CI)	*p* ^c)^	HR (95 % CI)	*p* ^c)^	HR (95 % CI)	*p* ^c)^
Location	Stomach	Reference	<0.001	Reference	0.002	Reference	<0.001	Reference	<0.001
	Small intestine	0.87 (0.78–0.97)		0.98 (0.87–1.10)		0.90 (0.78–1.03)		0.96 (0.83–1.11)	
	Other	1.31 (1.13–1.53)		1.30 (1.12–1.52)		1.54 (1.30–1.84)		1.46 (1.22–1.74)	
Size	<5 cm	Reference	<0.001	Reference	<0.001	Reference	<0.001	Reference	<0.001
	5 cm–9.9 cm	1.13 (0.97–1.32)		1.04 (0.89–1.21)		1.52 (1.23–1.89)		1.38 (1.12–1.72)	
	10 cm+	1.94 (1.68–2.25)		1.63 (1.40–1.89)		3.15 (2.58–3.85)		2.52 (2.05–3.08)	
	Unknown	2.36 (2.01–2.78)		1.49 (1.25–1.78)		3.72 (2.99–4.62)		2.00 (1.58–2.52)	
Metastatic disease	M0	Reference	<0.001	Reference	<0.001	Reference	<0.001	Reference	<0.001
	M1	2.84 (2.56–3.16)		2.03 (1.80–2.28)		3.69 (3.26–4.17)		2.42 (2.11–2.78)	
N stage	N−	Reference	<0.001	Reference	0.001	Reference	<0.001	Reference	0.001
	N+	2.28 (1.89–2.74)		1.47 (1.21–1.79)		2.73 (2.20–3.38)		1.55 (1.24–1.93)	
	NX	1.83 (1.63–2.05)		1.09 (0.96–1.24)		2.12 (1.85–2.42)		1.16 (1.00–1.36)	
Surgery of the	No surgery primary	Reference	<0.001	Reference	<0.001	Reference	<0.001	Reference	<0.001
primary	Surgery primary	0.33 (0.29–0.36)		0.49 (0.43–0.56)		0.27 (0.24–0.31)		0.44 (0.38–0.52)	
	Unknown	0.87 (0.36–2.09)		1.36 (0.56–3.31)		0.90 (0.34–2.42)		1.36 (0.50–3.67)	
Year	1998 to 2002	Reference	<0.001	Reference	<0.001	Reference	<0.001	Reference	<0.001
	2003 to 2005	0.79 (0.70–0.90)		0.76 (0.67–0.86)		0.76 (0.66–0.88)		0.73 (0.63–0.85)	
	2006 to 2008	0.67 (0.58–0.77)		0.61 (0.53–0.71)		0.66 (0.56–0.79)		0.63 (0.53–0.75)	
	2009 to 2011	0.63 (0.53–0.76)		0.62 (0.51–0.75)		0.55 (0.44–0.69)		0.55 (0.44–0.70)	
Gender	Male	Reference	<0.001	Reference	<0.001	Reference	0.001	Reference	<0.001
	Female	0.79 (0.72–0.88)		0.70 (0.63–0.78)		0.81 (0.72–0.92)		0.77 (0.68–0.88)	
Age	<50	Reference	<0.001	Reference	<0.001	Reference	<0.001	Reference	<0.001
	50–64	1.27 (1.08–1.50)		1.40 (1.19–1.66)		1.00 (0.83–1.19)		1.11 (0.92–1.33)	
	65–79	2.25 (1.92–2.63)		2.64 (2.25–3.10)		1.54 (1.29–1.83)		1.83 (1.53–2.18)	
	80+	4.92 (4.15–5.83)		5.64 (4.70–6.76)		2.91 (2.39–3.55)		3.19 (2.58–3.95)	
Ethnicity	Caucasian	Reference	0.001	Reference	0.002	Reference	0.013	Reference	0.058
	African-American	1.19 (1.06–1.35)		1.22 (1.07–1.39)		1.15 (0.99–1.34)		1.08 (0.92–1.27)	
	Other/Unknown	0.86 (0.74–1.01)		0.88 (0.76–1.03)		0.82 (0.68–1.00)		0.83 (0.68–1.00)	
Marital status	Married	Reference	<0.001	Reference	<0.001	Reference	<0.001	Reference	<0.001
	Single	1.18 (1.03–1.36)		1.38 (1.19–1.59)		1.29 (1.10–1.53)		1.40 (1.18–1.66)	
	Other/Unknown	1.48 (1.32–1.66)		1.19 (1.05–1.35)		1.44 (1.25–1.65)		1.22 (1.05–1.42)	

Hazard ratios (HR) with 95 % confidence intervals

^a^univariate Cox regression analysis

^b^multivariable Cox regression analysis full model including all covariates depicted in the table rows on the left

^c^likelihood ratio tests

### Trend analysis

**Fig. 6:**
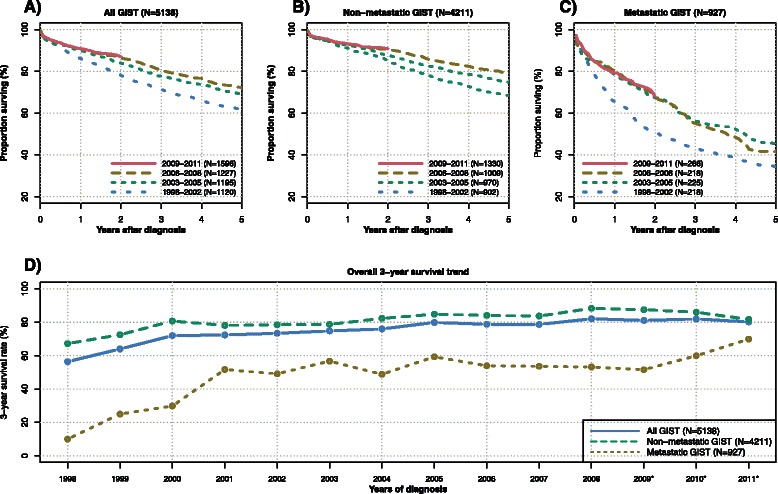
Trends in overall survival. Panel (**a**) to C display Kaplan-Meier curves for overall survival for all GIST (**a**), non-metastatic (**b**) and metastatic GIST (**c**) in four time intervals. The last interval from 2009 onwards is limited to two years of follow-up. Panel (**d**) displays the observed annual overall survival rates from 1998 to 2008 and the extrapolated survival rates for 2009 to 2011

Overall survival in four different time periods is displayed in Fig. 6 for the entire patient cohort (panel a), as well as for non-metastatic (panel b) and metastatic (panel c) GIST patients. There has been a significant improvement in overall survival over time (all GIST: P_Trend_ < 0.001, non-metastatic GIST: P_Trend_ = 0.001, and metastatic GIST: P_Trend_ = 0.013). The overall three-year survival for all GIST patients increased from 57.4 % (95 % CI: 48.3 to 68.2 %) in 1998 to 82.7 % (95 % CI: 79.1 to 86.3 %) in 2008. For non-metastatic GIST, the overall three-year survival increased from 68.5 % (95 % CI: 58.8 to 79.8 %) in 1998 to 88.6 % (95 % CI: 85.3 to 92.0 %) in 2008 and for metastatic GIST from 15.0 % (95 % CI: 5.3 to 42.6 %) in 1998 to 54.7 % (95 % CI: 44.4 to 67.3 %) in 2008. The annual percent change in three-year overall survival from 1999 to 2008 in all GIST patients was 11.9, 11.1, 0.6, 1.4, 1.9, 1.6, 4.9,−1.4,−0.1, and 4.1 %. In accordance with the hazard ratios and their confidence intervals for the year of diagnosis in the multivariable analysis (Table 2), most of the increase in the survival occurred in all sub-groups during the time before 2002. This is further depicted in panel d additionally demonstrating extrapolated estimates for the overall survival after 2008.

**Fig. 7:**
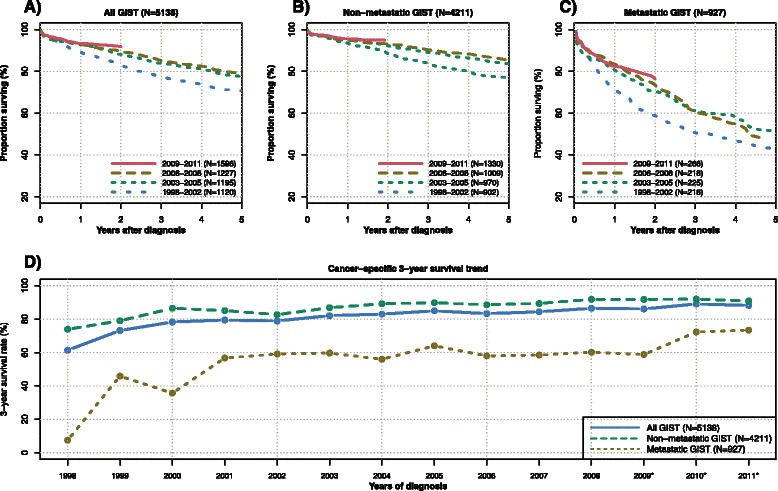
Trends in cancer-specific survival. Panel (**a**) to (**c**) display Kaplan-Meier curves for cancer-specific survival for all GIST (**a**), non-metastatic (**b**) and metastatic GIST (**c**) in four time intervals. The last interval from 2009 onwards is limited to two years of follow-up. Panel (**d**) displays the observed annual cancer-specific survival rates from 1998 to 2008 and the extrapolated survival rates for 2009 to 2011

Figure 7 displays the Kaplan-Meier curves for cancer-specific survival for all patients (panel a), for non-metastatic (panel b) and metastatic (panel c) GIST patients for the same time intervals. The cancer-specific survival significantly improved over time (all GIST: P_Trend_ < 0.001, non-metastatic GIST: P_Trend_ = 0.001, and metastatic GIST: P_Trend_ = 0.013). The three-year cancer-specific survival increased from 62.5 % (95 % CI: 53.4 to 73.2 %) in 1998 to 87.1 % (95 % CI: 83.9 to 90.3 %) in 2008 for all GIST patients, from 75.3 % (95 % CI: 66.1 to 85.9 %) in 1998 to 92.2 % (95 % CI: 89.4 to 95.1 %) in 2008 in non-metastatic GIST and from 15.0 % (95 % CI: 5.3 to 42.6 %) in 1998 to 61.9 % (95 % CI: 51.4 to 74.5 %) in 2008 for metastatic GIST patients. The annual percent change for the three-year cancer-specific survival from 1999 to 2008 in the entire cohort was 16.1, 6.5, 1.5,−0.8, 3.9, 1.1, 2.4,−2.0, 1.3, and 2.4 %. Hence, the improvement in cancer-specific survival occurred mainly before 2002.

## Discussion

This is the first population-based trend analysis of GIST patients over an 11-year time period. The present study provides compelling evidence of a statistically significant and clinically relevant overall and cancer-specific survival increase from 1998 to 2008, both in non-metastatic GIST as well as metastatic GIST. In addition to the well-known poor prognostic factors such as larger tumor size, nodal or distant metastases, and older age, we found that earlier time point of diagnosis, male gender, and single marital status are associated with worse overall and cancer-specific survival.

The present trend analysis was based on over 5000 GIST patients from the SEER registry. In this real-world analysis overall 3-year overall survival increased from 15 % in 1998 to 55 % in 2008 in metastatic GIST and from 68 to 89 % in patients with non-metastatic GIST. There are several reasons for this substantial improvement. First, a large increase in overall and cancer-specific survival was observed during the first 4 years of our analysis (1998–2001). This was prior to the FDA approval of imatinib. One explanation is that very low risk GIST may have been misclassified as leiomyoma and hence were not included into our analysis prior to the GIST consensus meeting of 2001 [14, 15]. Perez and colleagues showed a significant increase in reported GIST incidence from 1992 to 2002 based on SEER data, which is almost certainly related to reclassification of various tumors (e. g. leiomyoma) as GIST. [16] The inclusion of these tumors may have falsely increased the incidence and survival of GIST patients [17]. In the early years of our SEER analysis, the pivotal role of CD117 immunostaining was not systematically performed as previously pointed out by Tran and colleagues [18]. Hence, the incidence of GIST patients reported in SEER may be lower compared to studies, for which CD 117 staining was mandatory. Another explanation of the survival increase seen in the metastatic and non-metastatic group in the present analysis is stage migration. Indeed, PET scanning became a popular tool in the evaluation of GIST patients in the early and mid-2000’s, potentially leading to stage migration (Will Rogers phenomenon). Another explanation of the improved outcomes seen in the present investigation may the introduction of imatinib and other tyrosine kinase inhibitors in GIST treatment. There is no doubt that the advent of imatinib in treating GIST represents an important step forward in cancer care as this targeted therapy—already very successful in patients with chronic myeloid leukemia—was now being applied for the first time to a solid gastrointestinal cancer. Over the past decade, several randomized controlled trials investigating imatinib were performed demonstrating improved outcomes in patients with completely resected [3, 4] and metastatic GIST [5, 6]. While there are currently no other drugs than imatinib being used in non-metastatic GIST, several tyrosine kinase inhibitors have been associated with increased overall survival in patients with metastatic GIST. In addition to imatinib, which is used as a first line treatment, sunitinib [19] and regorafenib [20] have been evaluated in phase III randomized trials and resulted in an overall (sunitinib) and progression-free survival benefit (regorafenib) in second and third line treatment. Unfortunately, the use of tyrosine kinase inhibitors is not coded in the SEER database and hence an association between use of these systemic treatments and improved outcomes remains speculative.

Therefore, with more efficacious treatment options in advanced GIST patients, it is expected that overall and cancer-specific survival will continue to increase in the coming years as also shown in a data extrapolation in the present study (Fig. 5). In the adjuvant setting, the outcomes will most likely improve as well. In 2012, the German/Scandinavian study by Joensuu and colleagues provided compelling evidence that high-risk GIST patients have a better progression-free and overall survival with three years of adjuvant imatinib compared to only one year [4]. It is well known and also clearly seen in the German/Scandinavian trial that most recurrences occur within the first 12–24 months after stopping imatinib. Currently large randomized studies are undertaken to prove the hypothesis that 5 years of adjuvant imatinib treatment is superior to three years in the high-risk GIST subset. Selected patients with high-risk features (e. g. gastric GIST with very high mitotic count or non-gastric GIST with high mitotic count) may even benefit from life-long adjuvant imatinib treatment. However, this remains to be proven in well-designed and well-conducted trials as well as large cohort studies.

Both size and mitotic rate—the two best-known risk factors for recurrence—were evaluated in receiver operating curves in the present study. We identified a cut-off value of 8 cm and a mitotic rate of 5 per 50 high power fields (HPF) to be most predictive for cancer-specific survival. While the 5 mitosis per 50 HPF is a largely used cut-off value to risk stratify GISTs [21], a size cut-off value for worse prognosis set at 5 cm may be overly pessimistic (Fig. 4). Indeed, in an investigation by Woodall et al., which analysed GIST tumours based on SEER data from 1974 through 2004, a size cut-off of 7 cm was identified as an independent poor prognostic factor [22].

Moreover, in both univariate and multivariable analyses, a GIST size above 10 cm was associated with worse cancer-specific and overall survival while patients with GIST size between 5–10 cm had similar outcomes compared to those with a size of 5 cm and below. There is no doubt that a risk categorization of continuous biological variables such as size and mitotic rate is problematic. In this regard, prognostic contour maps as described by Joensuu et al. are helpful in assessing the risk of recurrence in GIST patients [23].

In the present analysis, patients with small bowel GIST had no worse overall and cancer-specific survival compared to patients with gastric GIST. This is opposed to other studies [24]. It is unclear why such a discrepancy occurs, however, may be due to different time periods in which the patients were enrolled in our study compared to the one by Gold and colleagues [24].

We would like to acknowledge the limitations of this study. The main drawback of this analysis is the lack of information on tyrosine kinase inhibitors used, data that cannot be ascertained in the SEER registry. Similarly, information about comorbidities, performance status, and information on site and number of metastases are not available in the SEER database. In addition, there is a relevant number of missing values for certain parameters e. g. mitotic rate, which was only systematically collected in the SEER database starting 2010. Despite these limitations, the present study has a variety of strengths. First, the population-based nature of the registry mirrors the real-world outcomes for GIST patients and is associated with a high degree of generalizability. It is key to evaluate to which extend advances in often highly selected patients in randomized controlled trials have translated into the overall patient population. Second, our study reports overall and cancer-specific survival data on a 11-years time period with extrapolation to a 14-years period. Third, the large sample size is associated with a high degree of power.

## Conclusion

In conclusion, larger tumor size, location other than stomach or small bowel, nodal or distant metastases, older age, earlier time point of diagnosis, male gender and single marital status are associated with significantly worse overall and cancer-specific survival. There has been a substantial increase in overall and cancer-specific survival from 1998 to 2008. It is anticipated that the current availability of different tyrosine kinase inhibitors in the advanced setting and better selection of high-risk patients benefitting from long-term adjuvant imatinib will continue to lead to a further improvement in patient outcomes.
